# Physical pain is common and associated with nonmedical prescription opioid use among people who inject drugs

**DOI:** 10.1186/s13011-017-0112-7

**Published:** 2017-05-30

**Authors:** Disa Dahlman, Alex H. Kral, Lynn Wenger, Anders Hakansson, Scott P. Novak

**Affiliations:** 1 Department of Clinical Sciences Lund, Division of Psychiatry, Lund University, Lund, Sweden; 2 Malmo Addiction Centre, Clinical Research Unit, Sodra Forstadsg. 35, plan 4, SE-205 02 Malmo, Sweden; 3 Behavioral and Urban Health Program, RTI International, San Francisco, USA; 4 Battelle Memorial Institute, Baltimore, MD USA

**Keywords:** Nonmedical use of prescription drugs, Opioids, Pain reliever, Physical pain, People who inject drugs

## Abstract

**Background:**

People who inject drugs (PWID) often have poor health and lack access to health care. The aim of this study was to examine whether PWID engage in self-treatment through nonmedical prescription opioid use (NMPOU). We describe the prevalence and features of self-reported physical pain and its association with NMPOU.

**Methods:**

PWID (*N* = 702) in San Francisco, California (age 18+) were recruited to complete interviewer administered surveys between 2011 and 2013. Multivariate logistic regression analysis was conducted to examine the associations among self-reported pain dimensions (past 24-h average pain, pain interference with functional domains) and NMPOU, controlling for age, sex, psychiatric illness, opioid substitution treatment, homelessness, street heroin use and unmet healthcare needs.

**Results:**

Almost half of the sample reported pain, based on self-reported measures in the 24 h before their interview. The most common pain locations were to their back and lower extremities. Past 24-h NMPOU was common (14.7%) and associated with past 24 h average pain intensity on a 10 point self-rating scale (adjusted odds ratio [AOR] = 2.15, 95% confidence interval [CI] 1.21–3.80), and past 24 h pain interference with general activity (AOR 1.82 [95% CI 1.04–3.21]), walking ability (AOR 2.52 [95% CI 1.37–4.63]), physical ability (AOR 2.01 [95% CI 1.16–3.45]), sleep (AOR 1.98 [95% CI 1.13–3.48]) and enjoyment of life (AOR 1.79 [95% CI 1.02–3.15]).

**Conclusion:**

Both pain and NMPOU are common among PWID, and highly correlated in this study. These findings suggest that greater efforts are needed to direct preventive health and services toward this population.

## Background

The nonmedical use of prescription opioids (NMPOU) has received considerable attention in the United States. Despite attention by the research and public policy communities, it is not surprising that the term “nonmedical use” has developed different definitions and usage within these different stakeholder communities [1]. It has been defined as ‘use without a prescription of the individual’s own or simply for the experience or feeling the drug caused’ [2]. However, it also has been described as misuse to get high, and as self-treatment for perceived physical or psychiatric problems [3, 4]. Most national surveys, such as the *National Survey on Drug Use and Health* (NSDUH) [5] and *Monitoring the Future* [6], combine both self-treatment and euphoric use into a single category. This definition masks important differences in terms of why individuals engage in NMPOU.

NMPOU has been described as a crisis by US public health authorities [7, 8] because of the dramatic increases in overdoses and substance abuse treatment admissions associated with opioid use [9]. Data from national studies indicate that NMPOU remains highly prevalent, despite recent efforts to control the prescriptions and diversion of medications to others [10, 11]. NSDUH estimates that in 2014, 1.6% of the U.S. population aged 12 years or older had been using prescription analgesics nonmedically in the past month [5]. These data also consistently show that opioids are the most common prescription drugs used nonmedically.

NMPOU has been linked to numerous adverse health-related consequences, largely due to its depressogenic effects on numerous biological systems, including the central nervous system causing somnolence or even unconsciousness and suppressing respiratory activity, and bowel and lower GI tract (e.g., opioid induced constipation). People who use prescription opioids nonmedically are sometimes also injecting drugs, which is critical from a public health standpoint, because of harms associated with injections such as venous scarring and infectious diseases [12, 13].

It is well documented that people who inject drugs (PWID) often lack stable employment and have inconsistent lines of income [14]. This economic instability means that PWID are often ineligible for government economic subsidies, including employer sponsored health insurance. PWID also experience unstable housing [15, 16] which complicates their ability to achieve a medical home that can provide consistent preventive and acute care. Overall, the highly unstable environment of PWID contributes to poor health and nutrition status, both of which may increase the risk of physical pain related to both chronic and acute conditions [17–20]. There is a longstanding tradition of research on documenting the prevalence of physical pain in general population surveys. Unfortunately, few studies have focused on physical pain in PWID. The limited studies suggest that chronic pain is highly prevalent. In studies by Tsui et al. [21] and Heimer et al. [22], one-third of PWID reported chronic pain. High prevalence of venous insufficiency, causing chronic leg pain, has been shown in PWID [23, 24].

A few large-scale studies have investigated the association among physical pain and NMPOU. Novak et al. [25] showed a positive linear correlation between the level of self-reported pain interference and the likelihood of past year NMPOU in the general North American population. The study further showed that self-reported pain was an independent risk factor for NMPOU, yet its effects were substantially modified by patterns of substance use. A survey of the general population in Sweden showed that more days with poor self-assessed physical health were significantly associated with NMPOU, but not with the nonmedical use of sedatives [26]. Besides these studies, research on NMPOU and its associations with pain is sparse, and studies are conducted mostly in outpatient populations receiving chronic opioid therapy. A meta-analysis by Fishbain et al. [27] showed that a diagnosis of drug abuse, drug dependence or drug addiction was present in 3%-19% of persons with chronic pain. Back pain and multiple pain complaints were factors identifying patients in chronic opioid therapy at high risk for misuse [28]. Other studies of patients with chronic pain showed no association between pain score and NMPOU [29, 30]. A recent longitudinal study by Martel et al. [31] showed that high level of pain was only weakly associated with opioid craving among patients with chronic pain.

A notable gap in the literature is that few studies have investigated the relationship between different dimensions of pain and NMPOU, particularly among high-risk populations who contend with high levels of pain such as PWID. There have been some studies that have explored rather course measures of self-reported pain. For example, Khosla et al. found a positive association between pain and a single measure of any type of nonmedical prescription drug use (NMPD) in American PWID [32]. The authors found that NMPD among PWID was significantly associated with self-reported bodily pain and pain interference with activity, and also with the hazardous use of alcohol, use of illicit substances, and active injection drug use. Misuse of prescription analgesics was the most common type of NMPD (17% reported opiate use in the past 6 months). However, a limitation of this prior work is that pain was assessed with a single item or that pain interference was concerning global functioning. To our knowledge, no studies have examined the relationship between distinct aspects of pain (e.g., location, duration and severity) and NMPOU.

The current paper provides a more refined examination between pain and NMPOU. A primary aim is to, in a sample consisting solely of PWID, describe the prevalence of recent physical pain, pain duration, pain intensity and pain interference. Then, a second aim is to characterize subgroups at differential impairment and functioning related to pain and investigate the association between NMPOU and pain-related factors.

## Methods

### Study sample and procedures

The study sample for this cross-sectional study comprised 706 PWID in San Francisco, California, with the data collected between November of 2011 and March of 2013. Potential participants were recruited from community settings using targeted sampling methods [33–35]. Three community field sites, located in neighborhoods near large populations of PWID, were utilized to conduct the interviews. Eligibility criteria included injection drug use in the past 30 days as verified by checking for recent signs of venipuncture, being 18 years of age or older, and the ability to provide informed consent [36]. Four persons were interviewed as part of the study, but whose data were later excluded because they answered on the survey that they had not injected drugs in the past 30 days. The survey was administered by a trained interviewer, and lasted between 45 and 60 min. Study participants received $20 (USD) for completing the interview. All study procedures were approved by the Institutional Review Board at RTI International.

### Instruments and measures

#### Outcome variable

The outcome variable was *NMPOU past 24 h*. The definition used the stem from the National Survey on Drug Use and Health, noting that “*The next questions are about drugs that are typically prescribed by physicians. We are interested in your use of these medications without a doctor's prescription and your use of these medications not as directed by your physician*.”

NMPOU past 24 h was assessed through the question “*When was the last time you used [prescription analgesic]?*” All persons who replied “within the past 24 h” (multiple choice question) for any of the following prescription analgesics (assessed separately) were recoded as ‘NMPOU past 24 h’: Vicodin, Oxycontin, Oxycodone, Percocet, Dilaudid, Fentanyl, Tramadol, Morphine, Embeda, Roxicodone, Opana, and other (Specify). We also included those who reported past 24 h nonmedical use of methadone, buprenorphine or Suboxone, i.e. those who replied “within the past 24 h” to any of the multiple choice questions “*When was the last time you used [Methadone/Buprenorphine or Suboxone] (not prescribed directly to you by a doctor or from a clinic, or the prescription was for you, but you took more than the Dr. prescribed)?*”

#### Explanatory variables

Explanatory variables were pain intensity, pain location, pain duration, and pain interference. Recent pain prevalence was measured using the yes/no question “*Have you had pain in the past 24 hours? Please include pain that might be masked by your drug or alcohol use.*” Pain intensity, and pain interference with general activity, mood, walking ability, physical ability, relationships, sleep and enjoyment of life were assessed using a modified version of the Brief Pain Inventory [37], which uses numeric scales where 0 was ‘no pain’ or ‘does not interfere’ and 10 was ‘pain as bad as you can imagine’ or ‘completely interferes.’ The Brief Pain Inventory, which has been widely used in studies measuring pain in substance-using populations [38–40], is validated for neuropathic pain [37] and is highly sensitive for pain assessment in patients in opioid substitution therapy [41]. The variables were recoded into three categories: 0 = no pain/interference (i.e. those who rated their pain/interference as 0 + those who didn’t have pain at all the past 24 h); 1 = pain/interference score from 1 to median; and 2 = pain/interference score above median. Since clinically relevant cut-off values for levels of pain and pain interference are difficult to estimate in this population, and the pain measurement values were not expected to follow a normal distribution, we dichotomized the values at the median. Median was calculated only for values 1–10 (0 excluded). The same procedure was conducted for pain duration. Missing data was recoded as ‘no pain reported (0)’ The rationale is that any person who skipped this item is likely doing so because they misinterpreted the item and felt they had no pain to report. Missing values did not exceed *n* = 3 for any of the variables.

For pain location, the study participants were provided a body chart that outlined 45 separate regions. The instructions of the inventory started with the instructions, “*Please look at the diagram and point to the area of your body that hurts the most.*” Several answers were allowed. We recoded the areas checked in the questions above into seven categories according to clinical relevance; head, neck and shoulders, back, upper extremities, lower extremities, abdomen, and genitals. Worst pain location was assessed through the question “*Which of the areas of your body is causing you the most pain?*” Responses according to the body chart were recoded into four mutually exclusive areas: head, neck/shoulders/back, extremities (upper and lower limbs), and abdomen/genitals. Where the response to this question was missing (*n* = 103), recoding into one of the four areas above was conducted if the subject had checked only one painful area for the question “Please look at the diagram and point to the area of your body that hurts the most.” Using this procedure, we were able to classify 99 individuals into one of four locations. If the subject had checked several painful areas (i.e. responses that could not be recoded into only one of four mutually exclusive areas), the subject was excluded from statistical analyses (*n* = 4).

#### Covariates

Based on the literature identifying associations between NMPOU and other substance use [25, 26, 29, 30, 42] and psychiatric disorders [26, 29, 42], we controlled all multivariate analyses for the following variables: Age at interview (left in its original metric) [28, 30]; biological sex at birth (male or female) [26, 30]; homelessness; lifetime psychiatric illness; use of street heroin; recent opioid substitution treatment (OST); and unmet healthcare needs.

Current homelessness was defined as a ‘yes’ to the question “*Do you consider yourself to be homeless*?”. Lifetime psychiatric disorder was defined as a ‘yes’ to the question “*As an adult, have you ever been diagnosed with a psychiatric illness (e.g., major depression, bipolar disorder)*?” Use of street heroin in the past 24 h was defined as replying “Within the past 24 h” to the question “*When was the last time you used [drug]?*” for any of the drugs speedball (heroin/cocaine, heroin/crack), goofball (heroin/methamphetamine) or heroin. Note that we did not discriminate routes of administration. OST in the past 30 days was defined as replying ‘yes’ to any of the questions “*Have you participated in methadone maintenance in the past 30 days?*” or “*In the past 30 days have you participated in buprenorphine (Suboxone) treatment?*”.

Unmet healthcare needs in the past 6 months was also included as a covariate. All subjects who replied ‘no’ to all three questions “*In the past 6 months did you need care for an urgent health problem such as an abscess, strep throat or the flu?*”, “*In the past 6 months did you need care for an ongoing health problem (e.g. high blood pressure, diabetes)?*”, and “*In the past 6 months did you need dental care?*” were recoded as ‘No need for healthcare in the past 6 months’. All subjects who reported need of any healthcare, and replied ‘no’ to either the question “*In the past 6 months, did you try to get [healthcare]?*” or the question “*In the past 6 months, have you received [healthcare]?*” were recoded as ‘Unmet health care need in the past 6 months’. All remaining subjects who were neither recoded as ‘No need for healthcare in the past 6 months’ nor ‘Unmet health care need in the past 6 months’ were recoded as ‘Met healthcare need in the past 6 months.’

The number of covariates in multivariate analysis was limited to one per 10 cases. Missing data values were recoded as ‘no’ (0). The number of missing values did not exceed *n* = 3 for any of the variables.

### Statistical analysis

We first started by estimating the bivariate associations between each of the 11 pain variables described above and NMPOU. We restricted the reporting window to the past 24 h. All variables were binary or categorical, and for statistical testing significance, we used unadjusted logistic regression analysis for binary outcomes. Multivariate logistic regression analysis was conducted with pain variables associated with NMPOU past 24 h at level *p* < 0.05 in bivariate analysis, adjusted for all pre-defined covariates. A correlation analysis was performed to prevent inclusion of explanatory variables and covariates with correlation 0.7 or more from the same analysis. *P*-values below 0.05 for a two-tailed test were considered statistically significant. All statistical analyses were performed in SPSS version 21.0 [43].

## Results

### Sample characteristics

Seven hundred and two PWID were included in the study. Twenty-one percent were female (Table 1). Mean age was 45.1 years (range 18–69). The most common street drugs used in the past 24 h were marijuana (36.8%), methamphetamine (27.6%) and heroin (24.6%). Fifteen percent (*n* = 103) reported NMPOU (including methadone and buprenorphine) in the past 24 h.

**Table 1:** Sample characteristics among people who inject drugs in San Francisco, for total sample (*N* = 702) and subjects reporting past 24 h pain (*n* = 335)

Characteristic	Pain past 24 h n (%)	Total sample n (%)	*P*-value
Mean age (range)	46.6 (19–69)	45.1 (18–69)	<0.001***
Sex			
Female	74 (22.1%)	147 (20.9%)	0.48
Male	261 (77.9%)	555 (79.1%)	
Race			
White	179 (53.4%)	379 (54.0%)	0.91
Black	86 (25.7%)	181 (25.8%)	
Hispanic	20 (6.0%)	46 (6.6%)	
Other	48 (14.3%)	91 (13.0%)	
*Missing*	*2 (0.6%)*	*5 (0.7%)*	
Homeless	208 (62.1%)	442 (63.0%)	0.65
Graduated from high school/got a GED	250 (74.6%)	509 (72.5%)	0.23
Ever diagnosed with a psychiatric illness*	207 (61.8%)	396 (56.4%)	0.01*
Health care need for an acute, chronic or dental problem in the past 6 months*			
No need	43 (12.8%)	122 (17.4%)	<0.01*
Met healthcare need	94 (28.1%)	204 (29.1%)	
Unmet healthcare need	198 (59.1%)	376 (53.6%)	
NMPOU past 24 h	53 (15.8%)	103 (14.7%)	0.41
NMPOU (except methadone and buprenorphine) past 24 h	50 (14.9%)	89 (12.7%)	0.09
Non-medical use of tranquilizers/sedatives past 24 h	23 (6.9%)	43 (6.1%)	0.44
Non-medical use of prescription stimulants past 24 h	2 (0.6%)	6 (0.9%)	0.48
Non-medical use of Methadone past 24 h	9 (2.7%)	23 (3.3%)	0.40
Non-medical use of buprenorphine/Suboxone past 24 h	0	1 (0.1%)	NA
Non-medical use of Phenergan past 24 h	3 (0.9%)	4 (0.6%)	0.27
Used Speedball (heroin/cocaine or heroin/crack) past 24 h	21 (6.3%)	42 (6.0%)	0.76
Used Goofball (heroin/methamphetamine) past 24 h	24 (7.2%)	40 (5.7%)	0.11
Used Crack or Rock Cocaine past 24 h	66 (19.7%)	139 (19.8%)	0.95
Used Powder Cocaine past 24 h	6 (1.8%)	15 (2.1%)	0.55
Used Methamphetamine past 24 h	92 (27.5%)	194 (27.6%)	0.92
Used Heroin past 24 h	80 (23.9%)	173 (24.6%)	0.65
Used Marijuana for non-medical reasons past 24 h	124 (37.0%)	258 (36.8%)	0.89
Used any type of street heroin past 24 h	93 (27.8%)	194 (27.6%)	0.94

*P*-value calculated with Pearson’s Chi-square test for all variables except age, where Student’s T-test was used

*p < 0.05

***p < 0.001

### Pain characteristics

Slightly less than half of the study participants, 47.7% (*n* = 335) reported that they had physical pain in the past 24 h (Table 2). Median pain duration was 36 months (interquartile range [IQR] 6–141 months). The most common pain locations were lower limbs (27.1%) and back (19.7%). Median average pain in the past 24 h was six on a 10-point scale (IQR 5–7). Approximately 40% of the sample reported past 24 h pain interference with the functional domains presented in Table 2.

**Table 2:** Pain prevalence and association with use of nonmedical prescription opioid use in the past 24 h among people who inject drugs in San Francisco (*N* = 702). Bivariate logistic regression analysis

Pain characteristics	Median (IQR)	Past 24 h NMPOU n (%)	Total sample n (%)	Unadjusted OR (95% CI)
Pain past 24 h^a^	NA	53 (51.5%)	335 (47.7%)	1.19 (0.78–1.81)
Pain duration in months^c^	36 (6–141)			
No pain (0)		50 (48.5%)	370 (52.7%)	1.00
Duration median or less (1–36)		27 (26.2%)	172 (24.5%)	1.19 (0.72–1.98)
Duration above median (37–776)		26 (25.2%)	160 (22.8%)	1.24 (0.74–2.08)
Pain single worst location (mutually exclusive)	NA			
No pain (0)		50 (49.0%)	367 (52.3%)	1.00
Head (1)		4 (3.9%)	17 (2.4%)	1.95 (0.61–6.22)
Neck/shoulder/back (2)		22 (21.6%)	122 (17.4%)	1.40 (0.81–2.42)
Extremities (3)		18 (17.6%)	158 (22.5%)	0.82 (0.46–1.45)
Abdomen/genitals (4)		8 (7.8%)	34 (4.8%)	1.95 (0.84–4.55)
*Missing values (multiple answers)*			*4 (0.6%)*	
Pain location (not mutually exclusive)	NA			
Head		10 (9.7%)	31 (4.4%)	NA4
Neck/shoulders		10 (9.7%)	77 (11.0%)	NA4
Back		27 (26.2%)	138 (19.7%)	NA4
Upper limbs		8 (7.8%)	67 (9.5%)	NA4
Lower limbs		28 (27.2%)	190 (27.1%)	NA4
Abdomen/genitals		10 (9.7%)	51 (7.3%)	NA4
Average pain past 24 h^c^	6 (5–7)			
No pain (0)		51 (49.5%)	375 (53.4%)	1.00
Pain median or less (1–6)		24 (23.3%)	201 (28.6%)	0.86 (0.51–1.45)
Pain above median (7–10)		28 (27.2%)	126 (17.9%)	1.82 (1.09–3.03)*
Pain interference with general activity past 24 h^c^	7 (5–9)			
No interference (0)		55 (53.4%)	412 (58.7%)	1.00
Interference median or less (1–7)		21 (20.4%)	165 (23.5%)	0.95 (0.55–1.62)
Interference above median (8–10)		27 (26.2%)	125 (17.8%)	1.79 (1.07–2.99)*
Pain interference with mood past 24 h^b^	7 (5–9)			
No interference (0)		54 (52.4%)	415 (59.1%)	1.00
Interference median or less (1–7)		26 (25.2%)	166 (23.6%)	1.24 (0.75–2.06)
Interference above median (8–10)		23 (22.3%)	121 (17.2%)	1.57 (0.92–2.68)
Pain interference with walking ability past 24 h^b^	8 (5–9)			
No interference (0)		55 (53.4%)	422 (60.1%)	1.00
Interference median or less (1–8)		25 (24.3%)	188 (26.8%)	1.02 (0.62–1.70)
Interference above median (9–10)		23 (22.3%)	92 (13.1%)	2.22 (1.28–3.86)**
Pain interference with physical ability past 24 h^b^	7 (5–9)			
No interference (0)		54 (52.4%)	404 (57.5%)	1.00
Interference median or less (1–7)		19 (18.4%)	161 (22.9%)	0.87 (0.50–1.52)
Interference above median (8–10)		30 (29.1%)	137 (19.5%)	1.82 (1.11–2.98)*
Pain interference with relationships past 24 h^c^	6 (3–8)			
No interference (0)		60 (58.3%)	465 (66.2%)	1.00
Interference median or less (1–6)		21 (20.4%)	122 (17.4%)	1.40 (0.82–2.42)
Interference above median (7–10)		22 (21.4%)	115 (16.4%)	1.60 (0.93–2.74)
Pain interference with sleep past 24 h^b^	7 (5–10)			
No interference (0)		56 (54.4%)	425 (60.5%)	1.00
Interference median or less (1–7)		19 (18.4%)	148 (21.1%)	0.97 (0.56–1.70)
Interference above median (8–10)		28 (27.2%)	129 (18.4%)	1.83 (1.10–3.03)*
Pain interference with enjoyment of life past 24 h^c^	7 (5–9)			
No interference (0)		54 (52.4%)	412 (58.7%)	1.00
Interference median or less (1–7)		23 (22.3%)	163 (23.2%)	1.09 (0.64–1.84)
Interference above median (8–10)		26 (25.2%)	127 (18.1%)	1.71 (1.02–2.86)*

*p < 0.05. **p < 0.005. *P*-value calculated with Wald Chi-square test

1. ^a^missing value recoded as “no pain”. 2. ^b^missing values recoded as “no pain”. 3. ^c^missing values recoded as “no pain”

4. Bivariate analysis was not conducted since pain locations were not mutually exclusive

### Associations between pain and NMPOU

In bivariate analysis, scores above median of pain intensity (average pain in past 24 h) and past 24 h level of pain interference with general activity, walking ability, physical ability, sleep and enjoyment of life were significantly and positively associated with NMPOU past 24 h (Table 2). Pain duration, pain location, and pain interference with mood and relationships were not statistically associated with NMPOU. After correlation analysis showing over 0.7 level correlation between all pain-related candidate variables, we conducted separate multivariate analyses assessing the association of each pain variable with NMPOU past 24 h. None of the covariates were excluded due to collinearity.

In multivariate analysis adjusted for age, sex, lifetime psychiatric diagnosis, homelessness, past 30 days OST, past 24 h use of street heroin and unmet healthcare needs in the past 6 months, NMPOU past 24 h was independently and positively associated with all the pain variable associated with NMPOU in bivariate analysis (Table 3).

**Table 3:** Multivariate logistic regression analysis of nonmedical prescription opioid use among people who inject drugs in San Francisco (*N* = 702). Outcome variable: NMPOU past 24 h

Characteristic	MODEL 1^a^ AOR (95% CI)	MODEL 2^b^ AOR (95% CI)	MODEL 3^c^ AOR (95% CI)	MODEL 4^d^ AOR (95% CI)	MODEL 5^e^ AOR (95% CI)	MODEL 6^f^ AOR (95% CI)
Average pain past 24 h						
Pain median or less	0.85 (0.49–1.47)	-	-	-	-	-
Pain above median	2.15 (1.21–3.80)*	-	-	-	-	-
Pain interference with general activity past 24 h						
Interference median or less	-	0.96 (0.54–1.70)	-	-	-	-
Interference above median	-	1.82 (1.04–3.21)*	-	-	-	-
Pain interference with walking ability past 24 h						
Interference median or less	-	-	1.05 (0.61–1.80)	-	-	-
Interference above median	-	-	2.52 (1.37–4.63)**	-	-	-
Pain interference with physical ability past 24 h						
Interference median or less	-	-	-	0.87 (0.48–1.57)	-	-
Interference above median	-	-	-	2.01 (1.16–3.45)*	-	-
Pain interference with sleep past 24 h						
Interference median or less	-	-	-	-	0.91 (0.50–1.65)	-
Interference above median	-	-	-	-	1.98 (1.13–3.48)*	-
Pain interference with enjoyment of life past 24 h						
Interference median or less	-	-	-	-	-	1.11 (0.63-1.94)
Interference above median	-	-	-	-	-	1.79 (1.02-3.15)*
Male sex	1.09 (0.64–1.86)	1.14 (0.67–1.93)	1.11 (0.65–1.90)	1.08 (0.63–1.84)	1.12 (0.66–1.90)	1.09 (0.64–1.86)
Age (continuous)	0.96 (0.94–0.98)***	0.96 (0.94–0.98)***	0.96 (0.94–0.98)***	0.96 (0.94–0.98)***	0.96 (0.94–0.98)***	0.96 (0.94–0.98)***
Lifetime psychiatric diagnosis	1.15 (0.72–1.84)	1.17 (0.73–1.87)	1.17 (0.73	1.18 (0.74–1.88)	1.09 (0.68–1.76)	1.18 (0.74–1.88)
Homeless	1.29 (0.78–2.13)	1.25 (0.75–2.06)	1.86)	1.25 (0.76–2.07)	1.24 (0.75–2.05)	1.24 (0.75–2.05)
OST past 30 days	1.64 (1.00–2.69)*	1.61 (0.98–2.63)	1.61 (0.98–2.64)	1.62 (0.99–2.66)	1.63 (0.99–2.67)	1.60 (0.98–2.62)
Street heroin use past 24 h	3.42 (2.17–5.39)***	3.33 (2.12–5.23)***	3.36 (2.14–5.30)***	3.31 (2.10–5.21)***	3.34 (2.12–5.25)***	3.30 (2.10–5.19)***
Healthcare need past 6 months^g^						
Met healthcare need	1.60 (0.69–3.70)	1.60 (0.69–3.70)	1.58 (0.68–3.66)	1.69 (0.73–3.91)	1.63 (0.70–3.78)	1.65 (0.71–3.81)
Unmet healthcare need	2.58 (1.20–5.55)*	2.58 (1.20–5.54)*	2.53 (1.18–5.66)*	2.72 (1.27–5.85)*	2.74 (1.28–5.90)*	2.65 (1.24–5.69)*

*p < 0.05, **p < 0.005, ***p < 0.001. *P*-value calculated with Wald Chi-square test

All models are adjusted for sex, age, lifetime psychiatric diagnosis, homelessness, opiate substitution treatment, use of street heroin and unmet healthcare needs

^a^Explanatory variable Pain intensity. Reference category is No pain

^b^Explanatory variable Pain interference with general activity. Reference category is No pain interference

^c^Explanatory variable Pain interference with walking. Reference category is No pain interference

^d^Explanatory variable Pain interference with physical ability. Reference category is No pain interference

^e^Explanatory variable Pain interference with sleep. Reference category is No pain interference

^f^Explanatory variable Pain interference with enjoyment of life. Reference category is No pain interference

^g^Healthcare need for an urgent/chronical/dental health problem. Reference category is No healthcare need past 6 months

## Discussion

The current study found that both pain and NMPOU were common among PWID, a high-risk population that traditionally lacks access to health care. The findings showing several independent cross-sectional associations between NMPOU and levels of pain and pain interference among PWID are novel and have important clinical implications.

Both recent and long-term pain was common in the study sample, which is similar to the sparse previous research on pain among PWID. Among HIV-positive patients, those who inject drugs have been shown to report more pain than those who do not inject drugs [44, 45]. Also, the prevalence of chronic pain is high among opioid dependent persons in opioid substitution treatment [46]. In one study, 37% reported chronic severe pain [40], and in another 61% reported chronic pain problems [47]. This high pain prevalence is not surprising, considering that homelessness, poverty and several potentially painful conditions such as dental problems [48], abscesses [49–51], other injection-related injuries [52] and chronic wounds [53] are common among PWID.

The primary contribution of this study was that we examined a diverse range of pain-related characteristics. While there was no statistically significant difference regarding NMPOU between subjects reporting no pain in the past 24 h and average pain median or below on the 10-point modified Brief Pain Inventory scale, subjects reporting average pain intensity above the median had more than doubled odds for NMPOU. Conversely, there was no association between NMPOU and pain duration. The association between pain intensity and NMPOU was similar to the results from surveys in the general American population study of non-institutionalized persons aged 18 or older showing a positive linear correlation between level of pain and past year NMPOU [25], but counter to previous research, which have not showed an association between pain and NMPOU in non-PWID cohorts consisting of U.S. veterans [29] and patients with chronic pain [30]. Given that we limited our measures to pain in the past 24 h, it is not surprising that we observed that pain was associated with NMPOU at the event-level over the previous day. Additional studies, perhaps using event-driven sampling like ecological momentary analysis, would be helpful in understanding the linkages between the onset of pain and NMPOU as a means to self-medicate pain.

We also observed that pain interference above median with general activity, walking ability, physical ability, sleep, and enjoyment of life was independently and positively associated with recent NMPOU.

Back pain and lower extremity pain was common in the study sample of PWID. This finding follows a study by Barry et al. [54] showing that chronic pain among persons seeking OST was most commonly located in back or legs. Lower extremity pain is common among PWID and homeless people, due to dermatological problems [55, 56], foot trauma and venous disorders [23, 24]. These pain locations are not notably different from what could be expected in the general population [57–60]. We did not find any significant association between pain location and NMPOU past 24 h, which is consistent with previous studies [29].

While the cross-sectional design of this study allows no interpretation of causality, our findings imply that PWID who use prescription pain relievers non-medically may be attempting to self-medicate or manage pain problems. Self-medication of pain, with heroin or prescription opioids, has been shown to be prevalent in 98% of PWID with moderate or extreme pain [61] and associated with being denied prescription analgesics. However, Heimer et al. [22] showed that four out of five PWID with chronic pain reported NMPOU before debut of their pain. In addition, up to three-quarters of patients with chronic non-cancer pain have been shown to have a lifetime history of substance use disorder [62]. Co-occurrence of pain and nonmedical substance use appears to be a complex matter, and future, longitudinal studies are necessary to assess causal relationships.

Covariates positively associated with NMPOU were younger age, use of street heroin, and unmet healthcare needs. Interestingly, recent use of street heroin was strongly associated with recent NMPOU in this study, while OST was not. Since all subjects in the study were PWID currently using drugs intravenously, this finding allows no interpretation regarding OST as a potential protective factor for avoiding NMPOU. However, future research assessing OST to diminish NMPOU would be of great clinical relevance. Worth noting is also that over half of the population had unmet healthcare needs in the past 6 months. We recommend future studies assessing unmet healthcare needs specifically as a predictor of NMPOU.

This study has several limitations that should be noted. All results were based on self-reports, and no structured clinical diagnosis or drug testing has been performed. We are unsure how the self-reported nature of the data on pain may bias our results, given that pain cannot be objectively measured. We believe that recall bias is kept to a minimum since the pain-related questions and the prescription and street drug questions were mostly about the past 24 h. The possibility of social desirability bias affecting the study participants’ reports of pain and drug use/NMPOU should, however, not be neglected. One additional limitation is that power was low to detect differences in pain as it related to NMPOU. We restricted our time-frame for most of our analyses to the past 24 h. This was done to boost our ability to link the timeframe in which pain could be self-mediated by prescription opioids. Unfortunately, a consequence of this decision was that there were a small number of cases that engaged in NMPOU during that timeframe. If we had expanded the window to the past 30 days, we would have gained statistical power by increasing the number of cases that endorsed NMPOU, but since most of the pain variables in the questionnaire were assessing past 24 h pain, we did not include past 30 days NMPOU data. Since temporality of pain and NMPOU could not assess in this cross-sectional study, reverse causality could not be excluded. It would thus be interesting to conduct longitudinal studies to examine whether individuals began NMPOU because of self-management, and then through habituation became tolerant of opioids. Neither physical comorbidity nor current psychiatric illness was possible to control for, which is an important limitation due to the large body of research data showing overlap between psychiatric morbidity and substance use [26, 29, 42].

The clinical implications of the present study are such that medical care is needed to address the high demand medical health needs of the PWID populations. Recently, several insurance companies in the United States have announced reductions in the patient coverage for opioid use as a means to reduce NMPOU [63, 64]. This complicates the situation for PWID, who already are disenfranchised from medical care and pain relief that could ease their pain and suffering, and there is a concern that prescribing restrictions might limit pain medication acquisition among legitimate pain patients. Previous research on pain management among vulnerable populations has identified inadequate analgesic therapy among 85% of patients with AIDS, and especially among AIDS-patients who were female, low-educated or PWID [65]. Voon et al. [66] showed that two-thirds of 462 PWID had ever been denied prescription analgesics, while 92% reported lifetime disability. Additional research is needed to determine whether or not these policies may have adverse impacts on PWID, including further removal from the main-stream medical system, or whether policies like the Affordable Health Care Act or similar reforms can serve to re-introduce those who have traditionally been excluded from the medical system.

## Conclusion

In conclusion, both pain and NMPOU were common among PWID, and pain intensity and pain interference were positively associated with NMPOU in this group. These findings implicate a strong need for improved physical healthcare among PWID. There is also a need for future longitudinal studies assessing the temporal nature of the associations between pain indicators and NMPOU, and evaluations of potential consequences of policies that restrict access to prescription opioids for high-risk populations including PWID.
